# Non-alcoholic fatty liver disease is associated with a higher risk of erectile dysfunction than alcoholic fatty liver disease

**DOI:** 10.3389/fmed.2025.1625498

**Published:** 2025-11-05

**Authors:** Hoi-Bor Chan, Sheng-You Su, Chun Lee, Chao-Yu Hsu

**Affiliations:** ^1^Department of Surgery, Ditmanson Medical Foundation, Chia-Yi Christian Hospital, Chia-Yi, Taiwan; ^2^Clinical Medicine Research Center, Ditmanson Medical Foundation, Chia-Yi Christian Hospital, Chia-Yi, Taiwan; ^3^Clinical Data Center, Ditmanson Medical Foundation, Chia-Yi Christian Hospital, Chia-Yi, Taiwan; ^4^Department of Medical Education, Ditmanson Medical Foundation, Chia-Yi Christian Hospital, Chia-Yi, Taiwan; ^5^Department of Artificial Intelligence and Healthcare Management, Central Taiwan University of Science and Technology, Taichung, Taiwan; ^6^Department of General Education, National Chin-Yi University of Technology, Taichung, Taiwan; ^7^Department of Senior Citizen Service Management, National Taichung University of Science and Technology, Taichung, Taiwan

**Keywords:** non-alcoholic fatty liver disease, alcoholic fatty liver disease, erectile dysfunction, TriNetX, fatty liver

## Abstract

**Objective:**

In this study, we examine the prevalence and risk of erectile dysfunction (ED) by conducting a comparative analysis between cohorts with alcoholic fatty liver disease (AFLD) and non-alcoholic fatty liver disease (NAFLD).

**Methods:**

This retrospective cohort study used the TriNetX database, including anonymized electronic health records from about 190 million patients globally. The study enrolled men aged ≥20 years diagnosed with AFLD or NAFLD between 2011 and 2019. Patients with liver cirrhosis or malignancy were excluded. Propensity score matching controlled for demographics and comorbidities. The primary outcome, incidence of ED, was analyzed at 1, 3 and 5 years using risk ratios (RR), odds ratios (OR) and hazard ratio (HR), ensuring balanced comparisons.

**Results:**

There were 9,066 AFLD and 431,064 NAFLD patients were enrolled before propensity score matching. Finally, following matching, 9,066 patients from each group were included for analysis. Within 1 year, NAFLD patients showed higher ED risk (2.394%) compared to AFLD patients (1.836%), with RR of 1.284 (1.052, 1.567), OR of 1.291 (1.054, 1.582) and HR of 1.263 (1.033, 1.544). At 3 years, NAFLD outcomes remained higher (5.228 vs. 4.169%), RR of 1.254 (1.099, 1.431), OR of 1.268 (1.104, 1.456) and HR of 1.221 (1.066, 1.397). By 5 years, NAFLD continued exhibiting greater risks (6.806 vs. 5.824%), RR of 1.169 (1.044, 1.308), OR of 1.181 (1.047, 1.331) and HR of 1.125 (1.002, 1.264). These findings demonstrate consistently elevated clinical risk in NAFLD patients vs. AFLD, highlighting the necessity of careful NAFLD monitoring.

**Conclusion:**

NAFLD demonstrates a significantly greater association with ED than AFLD. Clinicians should maintain heightened vigilance for ED when managing patients with NAFLD, particularly during the initial year following diagnosis.

## Introduction

Fatty liver disease is broadly classified into alcoholic and non-alcoholic subtypes, each characterized by distinct pathogenic mechanisms driving hepatic fat accumulation. Both etiologies represent significant contributors to the evolving global burden of hepatic disorders (1). A meta-analysis conducted by Liu et al. (2) reported a global prevalence of non-alcoholic fatty liver disease (NAFLD) of 29.38%, irrespective of the diagnostic modalities used. The prevalence of NAFLD has shown a marked upward trend over time, increasing from 27.94% during 2000–2010 to 31.63% in the period 2011–2021. In a separate study, Le et al. (3) estimated the global incidence of NAFLD at 46.13 cases per 1,000 person-years. The incidence was notably higher in males compared to females (59.43 vs. 36.71 per 1,000 person-years), and in individuals with obesity, who were approximately three times more likely to develop NAFLD than non-obese individuals (86.69 vs. 29.63 per 1,000 person-years). Country-specific analyses revealed that China exhibited a higher incidence of NAFLD compared to non-China regions, while Japan had a lower incidence compared to non-Japan regions. The authors emphasized the urgent need for public health strategies aimed at preventing NAFLD, particularly among high-risk groups such as males, individuals with obesity, and populations in regions with elevated incidence rates. NAFLD is increasingly recognized as a systemic disorder, with complications that extend beyond the liver and include erectile dysfunction (ED) (4).

The prevalence of alcohol use disorder is estimated at 5.1% globally, with the highest rates observed in the European Region and the Americas. Approximately 35% of individuals diagnosed with alcohol use disorder subsequently develop various manifestations of alcohol-associated liver disease (5). A meta-analysis conducted by Niu et al. (6), which synthesized data from 372 studies, determined that the worldwide prevalence of alcohol-related liver disease was 4.8%. Furthermore, their analysis revealed that alcoholic liver cirrhosis constituted the predominant clinical manifestation within the spectrum of alcohol-related liver disease, accounting for 32.9% of cases. In addition, alcohol not only damages the liver but also adversely affects other organ systems, including contributing to ED (7).

The global prevalence of ED has been reported to range widely from 3 to 76.5%, reflecting variations in study populations, methodologies, and definitions. ED is positively associated with cardiovascular disease, and affected individuals are at increased risk for adverse health outcomes. Specifically, men with ED exhibit a higher risk of all-cause mortality [odds ratio (OR): 1.26] and cardiovascular mortality (OR: 1.43) (8). The liver plays a fundamental role in the metabolism of numerous substances, including gonadal hormones and lipids. Hepatic disorders of a chronic nature frequently precipitate dysregulation in sex hormone homeostasis, glucose metabolism, and lipid processing–all of which constitute significant etiological factors for ED. Epidemiological investigations have documented that the prevalence of ED among male subjects with chronic liver disease ranges from 24.6 to 85.0%. Research conducted in Caucasian populations indicates that hepatic transplantation may ameliorate erectile function in patients with chronic liver diseases experiencing concomitant ED. This therapeutic response substantiates the pathophysiological relationship between chronic hepatic dysfunction and erectile impairment (9).

Both alcoholic fatty liver disease (AFLD) and NAFLD may contribute to the pathogenesis of ED. However, the differential stress burden contributing to ED between AFLD and NAFLD remains incompletely elucidated. To address this gap, the present study investigates the prevalence and risk of ED by performing a large-scale comparative analysis of cohorts with AFLD and NAFLD, thereby clarifying the differential impact of these two etiologies on sexual health outcomes.

## Materials and methods

### Data source

This study utilized data from TriNetX, a federated, cloud-based research network that harmonizes de-identified electronic health records contributed by more than 161 healthcare organizations worldwide. The network spans North America, Europe, Asia-Pacific, Latin America, and the Middle East, and includes diverse settings such as academic medical centers and community hospitals. Collectively, the platform provides access to over 190 million patient records, thereby supporting analyses that are geographically and demographically representative. The sampling frame consists of routinely collected clinical information, including demographic characteristics, diagnoses coded by International Classification of Diseases, Tenth Revision, Clinical Modification (ICD-10-CM), procedural data, laboratory test results, prescriptions, and longitudinal follow-up. All data undergo rigorous quality control and normalization before being integrated into the federated network. De-identification is performed according to Health Insurance Portability and Accountability Act (HIPAA) Privacy Rule standards (§164.514[b][1]), with attestation by an expert determination process, ensuring removal of protected health information. TriNetX has been widely applied in pharmacoepidemiology, outcomes research, and disease surveillance, with recent methodological evaluations confirming its validity, reproducibility, and global representativeness (10, 11).

### Study population

The current study enrolled men aged ≥20 years who received a new diagnosis of fatty liver disease between January 1, 2011, and December 31, 2019. Data were accessed from the TriNetX research platform on October 9, 2025. The study cohort was stratified into two distinct groups based on diagnostic criteria: AFLD (ICD-10-CM: K70.0) and NAFLD (ICD-10-CM: K76.0). The index date was defined as the date of the initial diagnosis of either AFLD or NAFLD. Patients with a history of liver cirrhosis (ICD-10-CM: K74.6 and K70.3) or malignant neoplasm of the liver and intrahepatic bile ducts (ICD-10-CM: C22) prior to the index date were excluded from the study. Baseline demographic and clinical characteristics were systematically extracted, including age, sex, and relevant comorbidities. Comorbid conditions identified were depression (ICD-10-CM: F32), diabetes mellitus (DM; ICD-10-CM: E08–E13), overweight and obesity (ICD-10-CM: E66), osteoarthritis (ICD-10-CM: M15–M19), and CKD (ICD-10-CM: N18), dyslipidemia (ICD-10-CM: E78), chronic ischemic heart disease (ICD-10-CM: I25), and testicular hypofunction (ICD-10-CM: E29.1). To minimize potential confounding and selection bias, propensity score matching was conducted utilizing the proprietary algorithm of the TriNetX platform, generating a matched cohort with a 1:1 ratio. After generating a propensity score for each patient, the system applies matching to identify comparable subsets. A greedy nearest-neighbor matching algorithm with a caliper width of 0.1 pooled standard deviations was used. Variables incorporated into the propensity score model included age, ethnicity, race, the aforementioned comorbidities and using of Statins (Anatomical Therapeutic Chemical [ATC]: C10AA) and Metformin (ATC: A10BA02). This rigorous matching approach ensured comparability between the two study groups and facilitated balanced baseline characteristics.

### Statistical analysis

The primary endpoint was the incidence of ED (ICD-10-CM: N52.9), evaluated at 1-, 3-, and 5-year intervals with comparative analyses conducted between the two cohorts. The quality of propensity score matching was evaluated using standardized differences (Std diff), with a Std diff threshold of <0.1 indicating acceptable balance in baseline characteristics between cohorts. Effect sizes were quantified using three complementary metrics: risk ratio (RR), OR and hazard ratio (HR). Each effect size was accompanied by 95% confidence intervals (CI). Statistical significance was determined using a *p*-value threshold of <0.05. The concurrent use of RR, OR and HR allowed for a more comprehensive evaluation of the relationship between fatty liver disease subtypes and the subsequent development of ED, thereby strengthening the analytical depth and interpretative reliability of the study findings.

## Results

**Figure 1:**
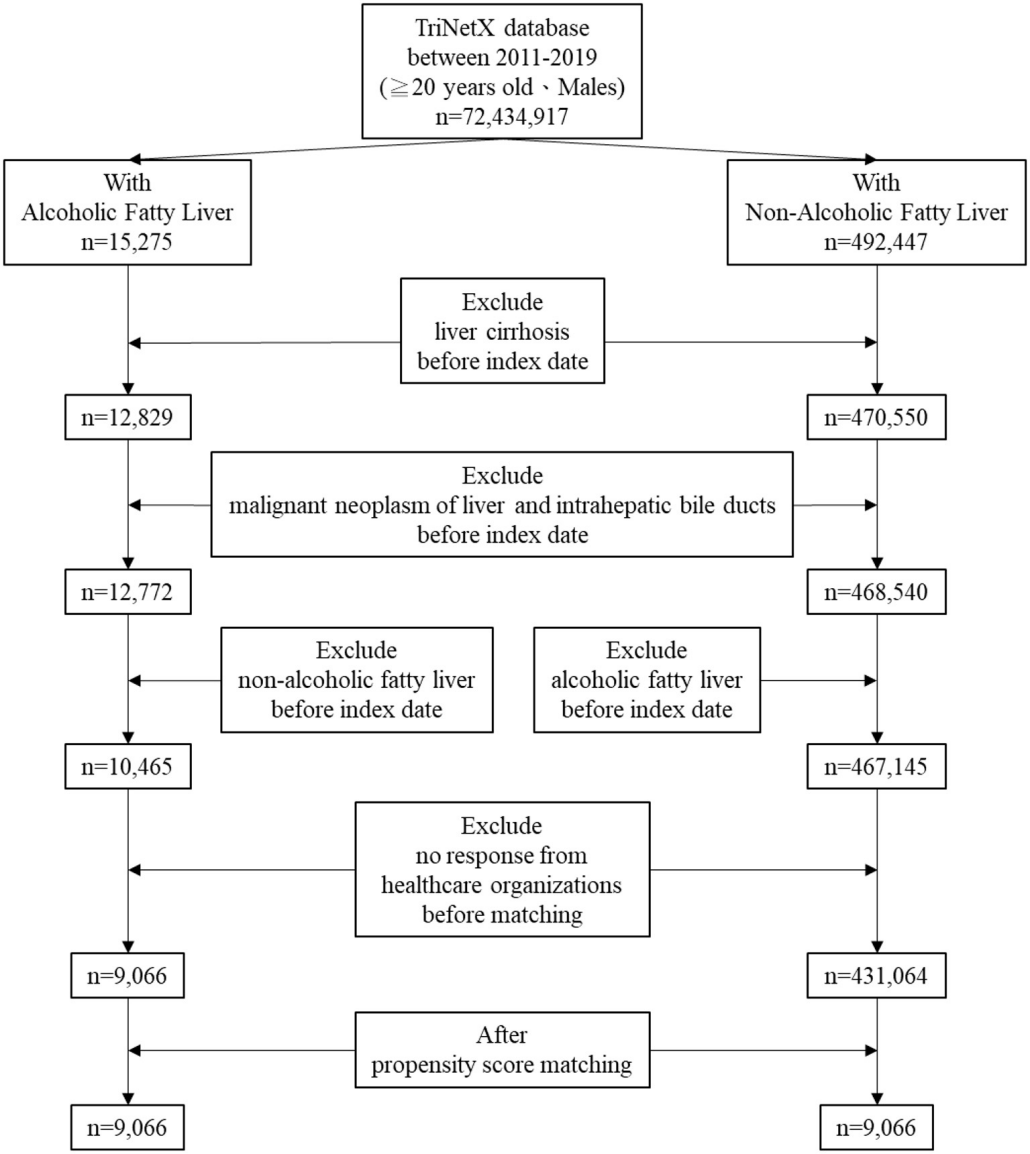
The flowchart of patients' selection.

Figure 1 illustrates the patient selection procedure using the TriNetX database from 2011 to 2019, including male patients aged 20 years or older, totaling 72,434,917 individuals. Table 1 presents the baseline characteristics of patients with NAFLD (*n* = 431,064) and AFLD (*n* = 9,066) before propensity score matching. The NAFLD cohort was slightly younger (50.5 ± 15.8 years) than the AFLD group (51.6 ± 12.9 years). NAFLD patients were more frequently Hispanic or White, whereas AFLD patients had a higher proportion of unknown ethnicity and race. Comorbidities such as DM, obesity/overweight, osteoarthritis, CKD, dyslipidemia, chronic ischemic heart disease and testicular hypofunction were markedly more prevalent among NAFLD patients, while depression was somewhat higher in AFLD. Use of statins and metformin was also significantly more common in NAFLD. Nearly all intergroup differences were statistically significant, with Std diff indicating notable baseline imbalance between cohorts prior to matching.

**Table 1:** Characteristics of non-alcoholic and alcoholic fatty liver disease cohorts before propensity score matching.

**Variables**	**Non-alcoholic fatty liver disease (*n*, %)**	**Alcoholic fatty liver disease (*n*, %)**	***p*-value**	**Std diff**
	**(*****n*** = **431,064)**	**(*****n*** = **9,066)**		
Age at index (mean ± SD)	50.5 ± 15.8	51.6 ± 12.9	<0.001	0.075
**Ethnicity**				
Hispanic or Latino	56,515 (13.1%)	685 (7.6%)	<0.001	0.183
Not Hispanic or Latino	271,749 (63.0%)	4,785 (52.8%)	<0.001	0.209
Unknown ethnicity	102,800 (23.8%)	3,596 (39.7%)	<0.001	0.345
**Race**				
White	292,279 (67.8%)	5,227 (57.7%)	<0.001	0.211
Asian	25,737 (6.0%)	330 (3.6%)	<0.001	0.109
Black or African American	32,064 (7.4%)	862 (9.5%)	<0.001	0.074
American Indian or Alaska Native	2,311 (0.5%)	57 (0.6%)	0.233	0.012
Native Hawaiian or Other Pacific Islander	3,566 (0.8%)	53 (0.6%)	0.011	0.029
Other race	16,592 (3.8%)	333 (3.7%)	0.388	0.009
Unknown race	58,515 (13.6%)	2,204 (24.3%)	<0.001	0.277
**Comorbidities**				
Depression	43,389 (10.1%)	1,079 (11.9%)	<0.001	0.059
Diabetes mellitus	85,671 (19.9%)	969 (10.7%)	<0.001	0.257
Overweight and obesity	84,633 (19.6%)	729 (8.0%)	<0.001	0.341
Osteoarthritis	51,192 (11.9%)	661 (7.3%)	<0.001	0.156
Chronic kidney disease	23,282 (5.4%)	263 (2.9%)	<0.001	0.126
Dyslipidemia	147,814 (34.3%)	1,856 (20.5%)	<0.001	0.314
Chronic ischemic heart disease	44,614 (10.4%)	603 (6.7%)	<0.001	0.133
Testicular hypofunction	11,633 (2.7%)	83 (0.9%)	<0.001	0.134
**Medication**				
Statins	89,620 (20.8%)	1,122 (12.4%)	<0.001	0.228
Metformin	44,663 (10.4%)	374 (4.1%)	<0.001	0.242

Std diff, standardized difference; SD, standard deviation.

Table 2 summarizes the baseline characteristics of NAFLD and AFLD cohorts after propensity score matching (*n* = 9,066 per group). Post-matching, the mean ages were nearly identical (51.5 ± 13.1 vs. 51.6 ± 12.9 years), and distributions of ethnicity and race were well-balanced, with Std diff below 0.01 for most variables. All comorbidities showed no statistically significant differences between cohorts. Similarly, the use of statins and metformin was comparable across groups. All *p*-values exceeded 0.05, and Std diff remained below 0.1, indicating successful balance of baseline characteristics. These results confirm that the matching procedure effectively minimized selection bias and ensured comparability between the NAFLD and AFLD cohorts.

**Table 2:** Characteristics of non-alcoholic and alcoholic fatty liver disease cohorts after propensity score matching.

**Variables**	**Non-alcoholic fatty liver disease (*n*, %)**	**Alcoholic fatty liver disease (*n*, %)**	***p*-value**	**Std diff**
	**(*****n*** = **9,066)**	**(*****n*** = **9,066)**		
Age at index (mean ± SD)	51.5 ± 13.1	51.6 ± 12.9	0.825	0.003
**Ethnicity**				
Hispanic or Latino	672 (7.4%)	685 (7.6%)	0.714	0.005
Not Hispanic or Latino	4,801 (53.0%)	4,785 (52.8%)	0.812	0.004
Unknown ethnicity	3,593 (39.6%)	3,596 (39.7%)	0.964	0.001
**Race**				
White	5,267 (58.1%)	5,227 (57.7%)	0.547	0.009
Asian	326 (3.6%)	330 (3.6%)	0.874	0.002
Black or African American	851 (9.4%)	862 (9.5%)	0.780	0.004
American Indian or Alaska Native	55 (0.6%)	57 (0.6%)	0.850	0.003
Native Hawaiian or Other Pacific Islander	52 (0.6%)	53 (0.6%)	0.922	0.001
Other race	331 (3.7%)	333 (3.7%)	0.937	0.001
Unknown race	2,184 (24.1%)	2,204 (24.3%)	0.729	0.005
**Comorbidities**				
Depression	1,030 (11.4%)	1,079 (11.9%)	0.256	0.017
Diabetes mellitus	939 (10.4%)	969 (10.7%)	0.468	0.011
Overweight and obesity	731 (8.1%)	729 (8.0%)	0.956	0.001
Osteoarthritis	644 (7.1%)	661 (7.3%)	0.625	0.007
Chronic kidney disease	226 (2.5%)	263 (2.9%)	0.090	0.025
Dyslipidemia	1,837 (20.3%)	1,856 (20.5%)	0.726	0.005
Chronic ischemic heart disease	551 (6.1%)	603 (6.7%)	0.114	0.023
Testicular hypofunction	76 (0.8%)	83 (0.9%)	0.577	0.008
**Medication**				
Statins	1,089 (12.0%)	1,122 (12.4%)	0.454	0.011
Metformin	378 (4.2%)	374 (4.1%)	0.882	0.002

Std diff, standardized difference; SD, standard deviation.

Table 3 presents the comparative risk of ED among patients with NAFLD (*n* = 450,890) and AFLD (*n* = 9,852) before propensity score matching. At the 1-year follow-up, the incidence of ED was 2.884% in NAFLD vs. 1.726% in AFLD (RR = 1.671, 95% CI = 1.439–1.942). The elevated risk persisted over time, with NAFLD patients showing higher ED incidence at 3 years (5.579 vs. 3.847%; RR = 1.450), and 5 years (7.435 vs. 5.369%; RR = 1.385). Corresponding OR and HR confirmed consistent and significant associations across all time intervals. Overall, these findings indicate that NAFLD patients experience a substantially greater risk of developing ED compared with those with AFLD.

**Table 3:** Risk analysis of erectile dysfunction within 1-, 3-, and 5-year periods before propensity score matching.

**Period**	**Patients with erectile dysfunction**		**Risk ratio (95% CI)**	**Odds ratio (95% CI)**	**Hazard ratio (95% CI)**
	**Non-alcoholic fatty liver disease (*****n*****, risk) (*****n*** = **450,890)**	**Alcoholic fatty liver disease (*****n*****, risk) (*****n*** = **9,852)**			
Within 1-year	13,004 (2.884%)	170 (1.726%)	1.671 (1.439, 1.942)	1.691 (1.452, 1.970)	1.591 (1.368, 1.851)
Within 3-year	25,154 (5.579%)	379 (3.847%)	1.450 (1.313, 1.602)	1.477 (1.332, 1.638)	1.361 (1.229, 1.506)
Within 5-year	33,523 (7.435%)	529 (5.369%)	1.385 (1.274, 1.505)	1.416 (1.296, 1.546)	1.281 (1.175, 1.395)

CI, confidence intervals.

**Figure 2:**
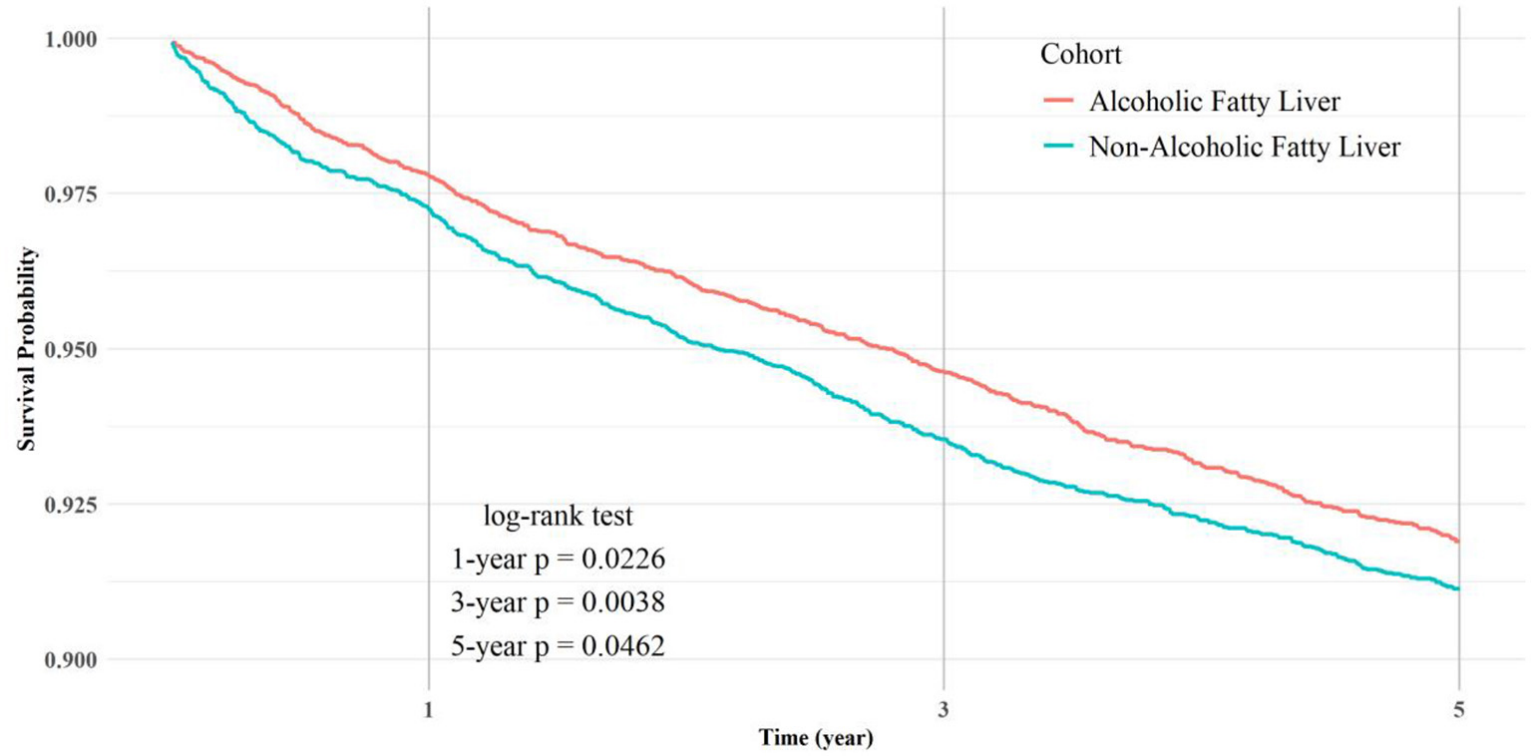
The survival probability of Kaplan-Meier analysis.

**Figure 3:**
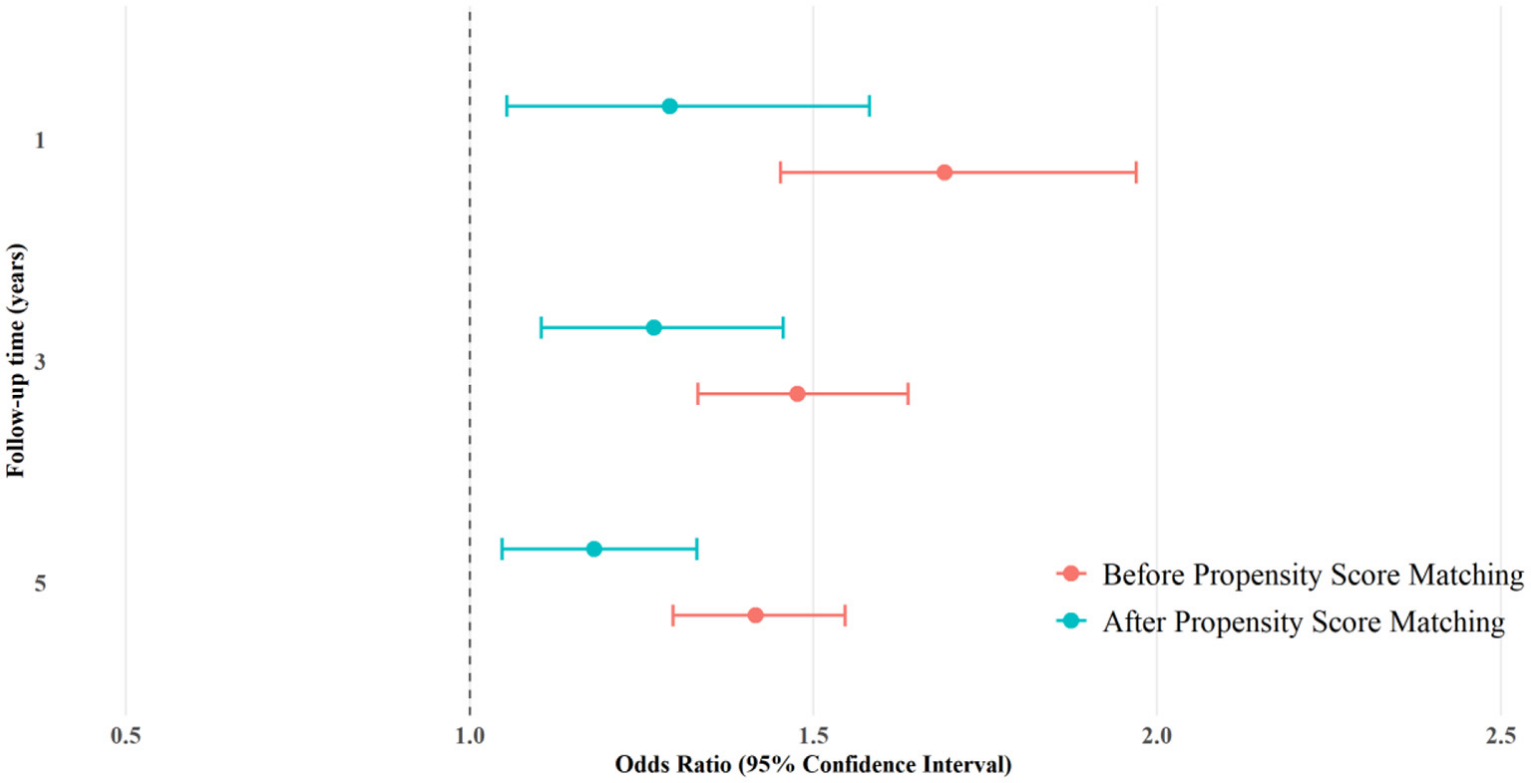
Forest plot of odds ratios (95% confidence intervals) for erectile dysfunction in patients with non-alcoholic fatty liver disease compared with alcoholic fatty liver disease before and after propensity score matching at 1-, 3-, and 5-year follow-up.

Table 4 presents the risk analysis of ED in NAFLD and AFLD cohorts after propensity score matching (*n* = 9,066 per group). Following covariate adjustment, NAFLD patients consistently demonstrated a higher incidence of ED across all follow-up periods. Within 1 year, the ED risk was 2.394% in NAFLD vs. 1.864% in AFLD (RR = 1.284, 95% CI = 1.052–1.567). This elevated risk persisted at 3 years (5.228 vs. 4.169%; RR = 1.254) and 5 years (6.806 vs. 5.824%; RR = 1.169). Corresponding OR and HR confirmed statistically significant differences throughout. The survival probabilities derived from Kaplan-Meier analysis are presented in Figure 2. Figure 3 shows a forest plot of OR (95% CI) for ED in NAFLD compared with AFLD before and after propensity score matching at 1-, 3-, and 5-year follow-up.

**Table 4:** Risk analysis of erectile dysfunction within 1-, 3-, and 5-year periods after propensity score matching.

**Period**	**Patients with erectile dysfunction**		**Risk ratio (95% CI)**	**Odds ratio (95% CI)**	**Hazard ratio (95% CI)**
	**Non-alcoholic fatty liver disease (*****n*****, risk) (*****n*** = **9,066)**	**Alcoholic fatty liver disease (*****n*****, risk) (*****n*** = **9,066)**			
Within 1-year	217 (2.394%)	169 (1.864%)	1.284 (1.052, 1.567)	1.291 (1.054, 1.582)	1.263 (1.033, 1.544)
Within 3-year	474 (5.228%)	378 (4.169%)	1.254 (1.099, 1.431)	1.268 (1.104, 1.456)	1.221 (1.066, 1.397)
Within 5-year	617 (6.806%)	528 (5.824%)	1.169 (1.044, 1.308)	1.181 (1.047, 1.331)	1.125 (1.002, 1.264)

CI, confidence intervals.

## Discussion

This study elucidates the burden of ED by comparing cohorts with AFLD and NAFLD using a large sample size and propensity score matching, which ensured comparability and balanced baseline characteristics between groups. Our findings reveal that patients with NAFLD face a significantly higher risk of developing ED than those with AFLD, with the risk difference being most pronounced within the first year after diagnosis.

In an earlier study, Cheng et al. (12) reported a negative association between regular alcohol consumption and ED. Their findings indicated that consuming one to seven alcoholic drinks per week did not affect the risk of ED; however, intake of 8 or more drinks per week was associated with a reduced risk. Subsequently, Li et al. (13) conducted a meta-analysis encompassing 46 studies with a total of 216,461 participants. They observed a J-shaped relationship between alcohol consumption and the risk of ED. Specifically, moderate alcohol consumption was associated with a reduced risk, whereas heavy alcohol intake did not show an association with ED risk. Recently, Mehta et al. (14) investigated the association between ED and the severity of alcohol dependence using the Severity of Alcohol Dependence Questionnaire and the International Index of Erectile Function. Their findings demonstrated that ED was prevalent among individuals with alcohol dependence syndrome, with severity of ED exhibiting a positive correlation with the severity of alcohol dependence. Comparative analysis of alcohol dependence severity and ED revealed that among severely alcohol-dependent patients, only 8.3% maintained normal erectile functioning. The distribution of moderate to severe ED across mild, moderate, and severe alcohol-dependent patients was 2.4, 8.0, and 66.7%, respectively. These results indicate a statistically significant elevation in ED severity among patients with severe alcohol dependence compared to those with mild or mild-moderate alcohol dependence. The causal relationship between alcohol consumption and ED is further elucidated by abstinence studies. Karunakaran et al. (15) documented that among 104 patients with alcohol use disorder who reported ED, 88.5% experienced improvement in erectile function following 3 months of alcohol abstinence. The researchers posited that the potential for ED improvement through alcohol abstinence could serve as a motivational factor in addiction treatment protocols, potentially enhancing therapeutic outcomes. Chronic alcohol consumption contributes to ED through multiple mechanisms. It impairs endothelial nitric oxide–mediated vasodilation, reducing penile blood flow (16). Ethanol enhances endothelin-1–induced contraction and oxidative stress in corpus cavernosum, promoting vasoconstriction (17). Additionally, long-term alcohol intake suppresses testosterone synthesis and alters sex hormone balance, leading to hypogonadism and reduced erectile function (18).

Duman et al. (19) identified a significant association between NAFLD and ED in their investigation of 40 patients. The researchers postulated that hepatic impairment may contribute to the pathophysiological mechanisms underlying ED in individuals with NAFLD. Similarly, Yilmaz et al. (20) reported a significantly greater prevalence of ED among patients diagnosed with NAFLD compared to those without NAFLD. Their findings indicated that patients with NAFLD exhibited a 2.92-fold higher risk of developing ED compared to patients without NAFLD. Nevertheless, it should be noted that their study population was relatively small, comprising only 106 patients with NAFLD and 40 patients without NAFLD, limiting the generalizability of these results. Youcheng et al. (21) subsequently conducted a more robust investigation with substantially greater statistical power. Employing the U.S. Fatty Liver Index (USFLI) with a diagnostic threshold of ≥30 for NAFLD, they evaluated 3,763 participants, among whom 29.1% reported ED. Their analysis revealed that compared to individuals in the lowest USFLI quartile (Q1: USFLI <10), subjects in the intermediate (Q2: 10 ≤ USFLI <30) and highest quartiles (Q3: USFLI ≥30, consistent with NAFLD) demonstrated adjusted OR for ED of 1.84 and 2.18, respectively. These findings substantiate a positive correlation between NAFLD severity and ED risk, establishing a dose-response relationship between these clinical entities. NAFLD contributes to ED through metabolic, hormonal, and vascular mechanisms. NAFLD is closely linked to metabolic syndrome, systemic inflammation, and endothelial dysfunction, all of which impair penile vascular response (22). Clinical studies show that insulin resistance and low testosterone are key predictors of ED among NAFLD patients, suggesting endocrine and metabolic dysregulation (4). Furthermore, reduced nitric oxide and adropin levels compromise endothelial integrity and vascular relaxation, highlighting the critical role of impaired nitric oxide signaling and energy metabolism in the pathogenesis of NAFLD-related ED (23).

Multiple established risk factors for ED have been documented in the literature, including depression (24), DM (25), obesity (26), and CKD (27). Our finding that NAFLD confers a higher risk of ED than AFLD is biologically plausible. NAFLD is tightly linked to systemic insulin resistance, visceral adiposity, atherogenic dyslipidemia, and chronic low-grade inflammation, each of which contributes to endothelial dysfunction and reduced nitric-oxide bioavailability—central mechanisms in ED pathogenesis. In addition, NAFLD has been associated with hypogonadism, oxidative stress, and microvascular rarefaction, which can further impair penile hemodynamics. Collectively, these pathways provide a coherent explanation for the higher ED burden observed in NAFLD, even after balancing major confounders via propensity score matching. Any apparent association between alcohol and health outcomes is often nonlinear (U/J-shaped), pattern-dependent (regular light intake vs. binge drinking), and vulnerable to residual confounding (e.g., socioeconomic status, diet, physical activity). Moreover, alcohol exposure exerts direct hepatotoxic effects, promotes oxidative injury, and disrupts the gut–liver axis. Accordingly, our findings should not be interpreted as endorsing alcohol use to reduce ED risk; rather, they suggest that NAFLD-related metabolic derangements are the predominant drivers of ED in this comparison. These findings underscore the importance of considering sexual health outcomes in the clinical evaluation and treatment planning for patients with fatty liver disease, particularly those with the non-alcoholic variant.

This retrospective study utilizing the TrinetX database presents several limitations that warrant acknowledgment. First, our study definitions for NAFLD and ED were derived exclusively from ICD-10-CM codes, without corroborating imaging findings or laboratory parameters. Although this approach is consistent with large-scale real-world database research, it may introduce misclassification bias and restrict interpretation regarding disease severity. Second, the significantly smaller number of AFLD patients compared with NAFLD may reflect differences in diagnostic coding practices, disease recognition, or healthcare-seeking behavior, thereby introducing potential selection bias. Moreover, as all patients were identified from healthcare systems contributing data to the TriNetX network, individuals without medical encounters were not captured, which may further limit the representativeness of the study population. Third, although we report 1-, 3-, and 5-year risks, follow-up duration was not uniformly available for all patients. Differential loss to follow-up at various time points may introduce temporal bias and potentially lead to underestimation of long-term risk. Fourth, although multiple known confounders were controlled for through propensity score matching, residual confounding cannot be fully excluded. Information on lifestyle behaviors and socioeconomic status was unavailable in the TriNetX database, despite the established confounding impact of smoking on erectile function (28, 29). Finally, this study relied on the TriNetX platform's built-in greedy nearest-neighbor matching algorithm without performing sensitivity analyses for multiple matching strategies or parameters. Future research should incorporate these analyses to further validate the robustness of the findings. Notwithstanding these limitations, our large-scale analysis indicates that NAFLD confers a higher risk of ED than AFLD. These findings offer important clinical implications for patient management and lay the groundwork for future interventional research.

## Conclusion

NAFLD demonstrates a significantly greater association with ED than AFLD. Clinicians should maintain heightened vigilance for ED when managing patients with NAFLD, particularly during the initial year following diagnosis. Future studies are warranted to elucidate the underlying mechanisms and to assess whether targeted metabolic interventions can mitigate this risk.

## Data Availability

The datasets presented in this article are not readily available because this study used de-identified, aggregate-level data from the TriNetX platform. Due to data use agreements and privacy regulations, raw patient-level data cannot be shared. All analyses, including cohort matching and outcome calculations, were performed within the TriNetX platform using built-in tools, limiting external replication. Requests to access the datasets should be directed to https://trinetx.com.
